# Case report: Metagenomic next-generation sequencing for the diagnosis of rare *Nocardia aobensis* infection in a patient with immune thrombocytopenia

**DOI:** 10.3389/fmed.2024.1425655

**Published:** 2024-11-06

**Authors:** Xiaocui Liang, Xiaoyu Liu, Zhimin Huang, Fei Qiu, Yini Jiang, Chunhong Li, Zhenfeng Deng, Jinyu Wu

**Affiliations:** ^1^Infection Diagnosis Center, Guangxi KingMed Diagnostics, Nanning, China; ^2^Department of Rheumatology, The First Affiliated Hospital of Guangxi University of Chinese Medicine, Nanning, China

**Keywords:** *Nocardia aobensis*, nocardiosis, metagenomic next-generation sequencing, diagnosis, case report

## Abstract

**Background:**

Nocardiosis poses a diagnostic challenge due to its rarity in clinical practice, non-specific clinical symptoms and imaging features, and the limitations of traditional detection methods. *Nocardia aobensis* (*N. aobensis*) is rarely detected in clinical samples. Metagenomic next-generation sequencing (mNGS) offers significant advantages over traditional methods for rapid and accurate diagnosis of infectious diseases, especially for rare pathogens.

**Case presentation:**

A 52-year-old woman with a history of immune thrombocytopenia for over 2 years was hospitalized for recurrent fever and cough lasting for 10 days. Her initial diagnosis on admission was community-acquired pneumonia, based on chest computed tomography findings of lung inflammation lesion. Empirical treatment with moxifloxacin and trimethoprim-sulfamethoxazole (TMP-SMZ) was initiated. However, her condition failed to improve significantly even after 1 week of treatment. Bronchoalveolar lavage fluid (BALF) subjected to mNGS revealed the presence of *N. aobensis*, resulting in a diagnosis of pulmonary nocardiosis caused by *N. aobensis*. This diagnosis was also supported by Sanger sequencing of the BALF. After adjusting the antibiotic regimen to include TMP-SMZ in combination with imipenem, the patient’s condition significantly improved. She was finally discharged with instructions to continue oral treatment with TMP-SMZ and linezolid for 6 months. The patient’s first follow-up 1 month after discharge showed good treatment outcomes but with obvious side effects of the drugs. Consequently, the antibiotic regimen was changed to doxycycline, and the patient continued to improve.

**Conclusion:**

We report the first detailed case of pulmonary nocardiosis caused by *N. aobensis* diagnosed by mNGS. mNGS could be an effective method that facilitates early diagnosis and timely decision-making for the treatment of nocardiosis, especially in cases that involve rare pathogens.

## Introduction

*Nocardia* is a genus of gram-positive, aerobic, branching filamentous bacteria, which is widely found in soil, decaying vegetation, and aquatic environments. Thus far, more than 100 species of *Nocardia* have been identified, of which 55 have been recognized as human pathogens (1, 2). *Nocardia* species are regarded as opportunistic pathogens that seldom cause infections in immunocompetent people but predominantly affecting patients with immunocompromised states (3). They can affect many organs such as the lungs, skin, joints, and central nervous system. Pulmonary nocardiosis is the most common manifestation of nocardiosis (4). The clinical symptoms and radiological presentations of pulmonary nocardiosis are non-specific, and the clinical diagnosis depends on microbial culture. However, this traditional culture method has low sensitivity and time-consuming. Therefore, the diagnosis of pulmonary nocardiosis is often missed or delayed, which contributes to a significantly high mortality rate (5, 6). Although PCR and immunology techniques are also used as conventional methods for the identification of pathogens, both of them are limited to specific pathogens, and prior knowledge of the suspected pathogens is necessary. Nocardiosis is uncommon in clinical practice, and it is usually be overlooked or unexpected by clinicians. Consequently, a rapid, accurate, and reliable method for identifying *Nocardia* in clinical samples is of paramount importance to assist clinicians to make timely diagnoses and select appropriate antimicrobial therapy, which can improve patient prognosis.

Metagenomic next-generation sequencing (mNGS) is a culture-independent high-throughput sequencing technology that can quickly and untargeted detect all pathogenic microorganisms, including viruses, bacteria, fungi, and parasites, in clinical samples with an unbiased manner. Recent studies have shown that mNGS offers superior diagnostic value compared to conventional methods for diagnosing nocardiosis, not only in accurately identifying *Nocardia* species but also in substantially shortening the detection turnaround time (7). The distribution of *Nocardia* species varies geographically; however, *Nocardia farcinica*, *Nocardia cyriacigeorgica*, *Nocardia brasiliensis*, and *Nocardia abscessus*, possibly in a different order, are the four most common species isolated from clinical samples (8). *Nocardia aobensis* (*N. aobensis*) is rarely detected in clinical samples, and few infections with this pathogen have been reported. Here, we present the first case of pulmonary nocardiosis due to *N. aobensis* diagnosed by mNGS with specific description.

## Case presentation

**Figure 1:**
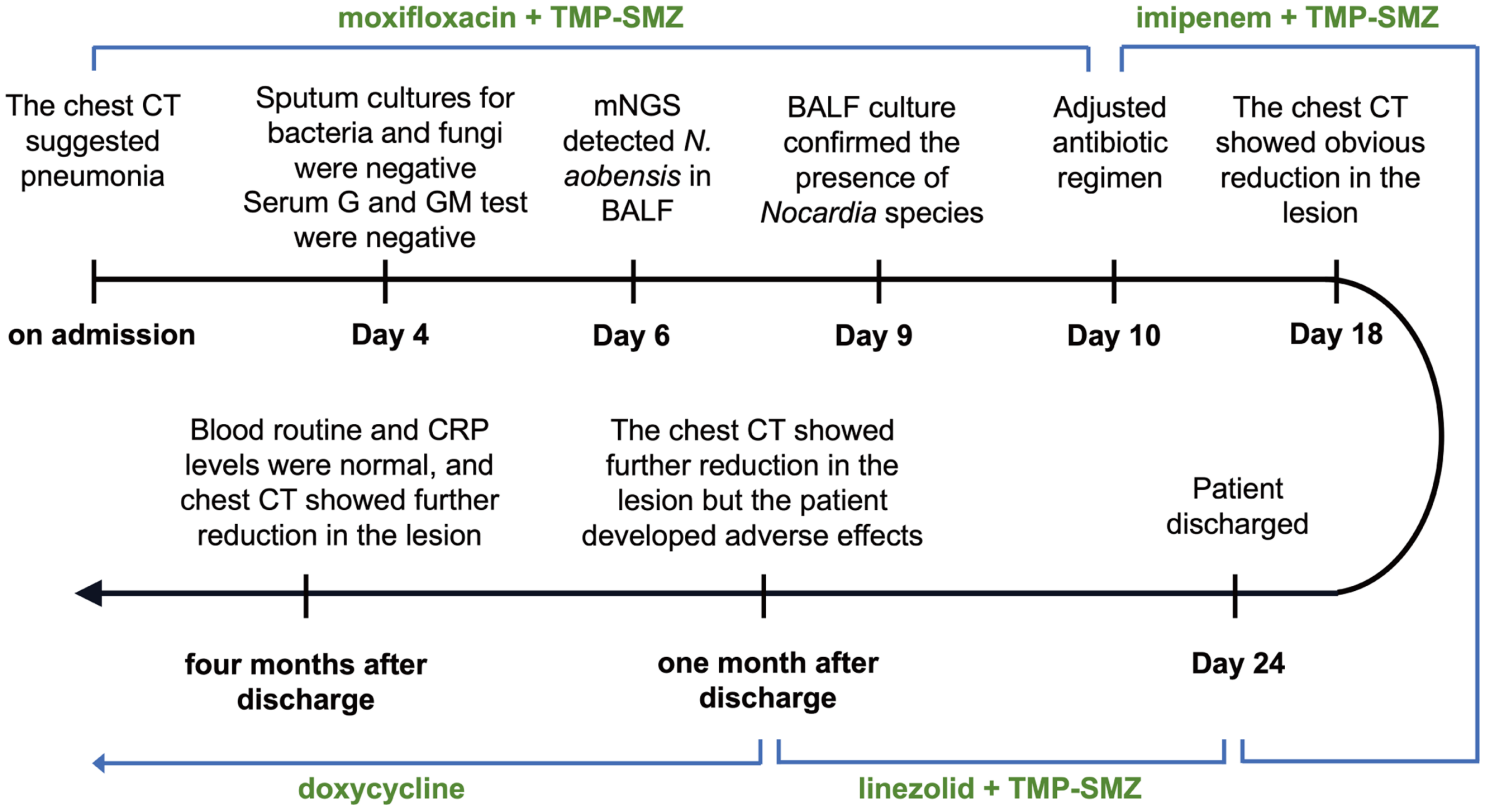
Timeline of the hospitalization and treatment course.

A 52-year-old woman was admitted to The First Affiliated Hospital of Guangxi University of Chinese Medicine on July 13, 2023, with seeking further treatment for recurrent fever that had started 10 days ago. The timeline of the hospitalization and treatment course is shown in Figure 1, and the case progress record in detail is reported as follows.

### Patient information

The patient previously worked as a farm laborer who currently retired and had been recuperating at home. Her fever started 10 days ago was irregular, reaching up to 38.5°C, accompanied by a cough producing a small amount of white sputum. There were no symptoms of chest tightness, palpitations, abdominal pain, or diarrhea. Her condition had not shown significant improvement without specific treatment. She had a history of immune thrombocytopenia for over 2 years and had been on long-term treatment with steroids and immunosuppressants. Her medication dosage had been adjusted multiple times based on her condition. Her previous medications included methotrexate, azathioprine, and baricitinib, whereas her current medication regimen consisted of methylprednisolone (20 mg qd) and cyclosporine (25 mg per tablet, taken as three tablets in the morning and two in the afternoon). Her medical history included hypertension, hyperlipidemia, chronic gastritis, multifocal hepatic hemangiomas, a left kidney cyst, herpes zoster, and osteoporosis. She denied any other significant contact history or family medical history.

### Physical examination

On admission, the patient’s vital signs were stable, with a temperature of 36.2°C, pulse rate of 90 beats/min, respiratory rate of 20 breaths/min, and blood pressure of 125/82 mmHg. Physical examination revealed coarse breathing sounds, with faint crackles in both lungs. Petechiae were observed on the right upper arm, and there was mild pitting edema in both lower limbs. Further inquiries revealed no evidence suggestive of coagulation function abnormalities, such as hematochezia, hematuria, or gingival bleeding.

### Laboratory and imaging examinations

**Figure 2:**
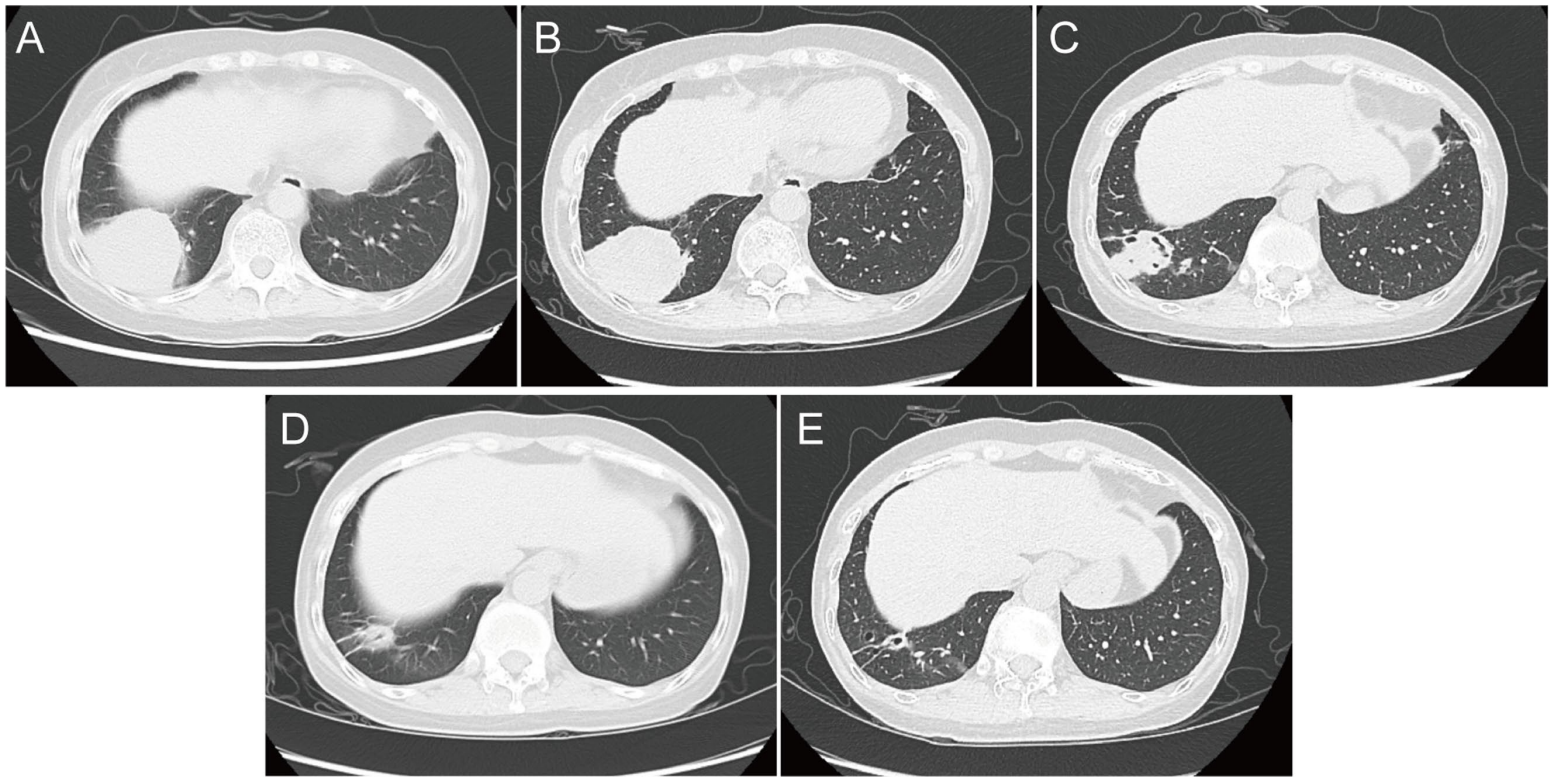
Chest computed tomography image. **(A)** At admission; **(B)** on Day 7; **(C)** on Day 18; **(D)** at follow-up 1 month after discharge; and **(E)** at follow-up 4 months after discharge.

Laboratory examinations revealed an elevated white blood cell count (10.86 × 10^9^/L; normal range: 3.5–9.5 × 10^9^/L), with an elevated neutrophil percentage (89.8%; normal range: 40–75%). The platelet count was within the normal range at 205 × 10^9^/L. The immunoglobulin-G levels were reduced at 6.120 g/L (normal range: 8.6–17.4 g/L), and both erythrocyte sedimentation rate (ESR) and C-reactive protein (CRP) levels were elevated, with values of 49 mm/h (normal range: 0–20 mm/h) and 86.4 mg/L (normal range: 0–6 mg/L), respectively. Chest computed tomography (CT) revealed a round high-density shadow in the lower lobe of the right lung, suggestive of inflammatory lesion. A solid nodule was also detected beneath the pleura in the upper lobe apical posterior segment of the left lung (Figure 2A).

### Microbiological examinations

**Figure 3:**
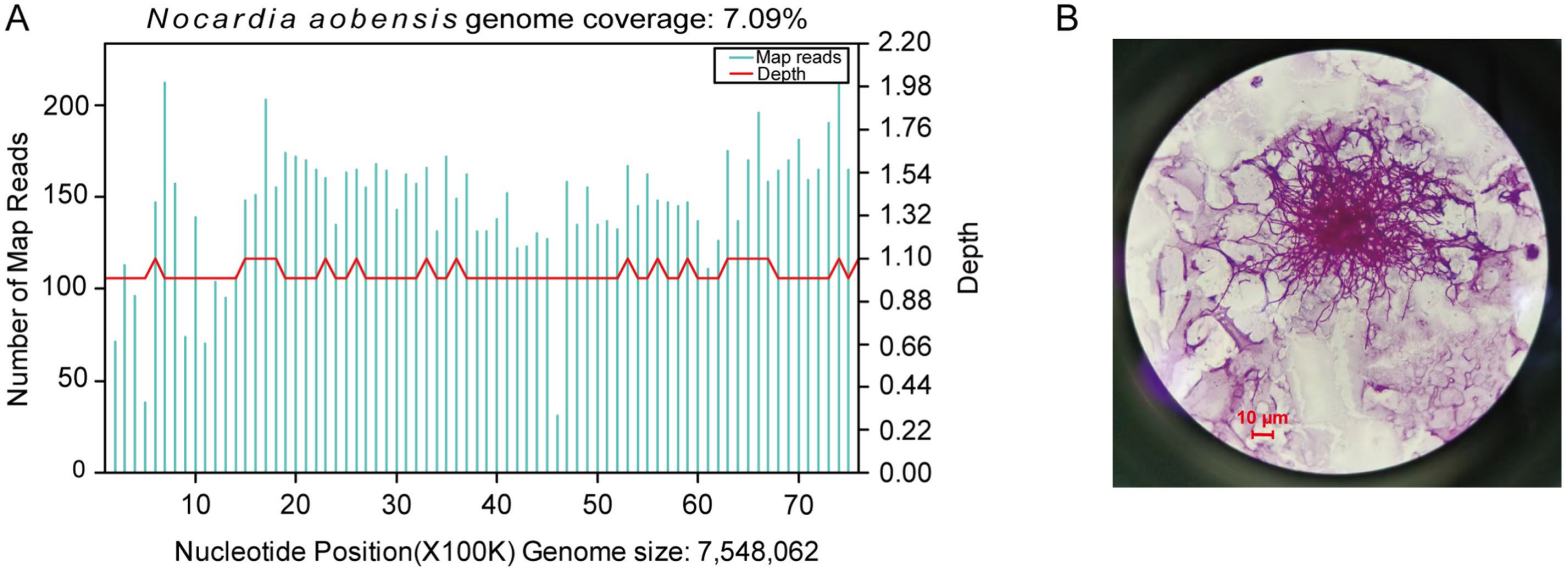
Diagnostic tests on bronchoalveolar lavage fluid. **(A)** Metagenomic next-generation sequencing of the BALF reported 9,806 reads of *Nocardia aobensis*, with a genome coverage of 7.09%. **(B)** Weak acid-fast staining of BALF showed positive result (×1,000).

On admission, the sputum specimens of the patient were submitted for bacterial and fungal culture. Gram staining of the sputum revealed the presence of gram-positive cocci (+++) and gram-positive bacilli (+). The acid-fast staining for tuberculosis of sputum was negative. On Day 4 after admission, bacterial and fungal cultures of the sputum were negative. In addition, tests for fungal infections, specifically serum aspergillosis galactomannan and (1,3)-*β*-d-glucan tests, were also negative. On Day 5, bronchoalveolar lavage fluid (BALF) was collected and subjected to mNGS and microbiological culture. Gram staining of BALF showed a few gram-positive cocci and a small number of gram-positive bacilli. On Day 6, mNGS results identified *N. aobensis* with a sequence count of 9,806 reads, genome coverage of 7.09%, and a relative abundance of 82.48% (Figure 3A). No other pathogenic microorganisms were identified. These sequences were submitted to the SRA database at NCBI with the accession number PRJNA1154608.^1^ Further testing of the BALF using Sanger sequencing confirmed the authenticity of *N. aobensis*. And the weak acid-fast staining of BALF yielded a positive result, which was consistent with the morphological features of *Nocardia* (Figure 3B). On Day 9, the BALF culture confirmed the presence of *Nocardia* species (++), while the fungal culture was negative.

### Treatment course

Upon admission, the patient was initially diagnosed with a fever of unknown origin and community-acquired pneumonia. The inflammatory lesion on chest CT image, suggestive of pneumonia, prompted the initiation of empirical antibiotic therapy with moxifloxacin in combination with trimethoprim-sulfamethoxazole (TMP-SMZ). The patient experienced no significant fever during hospitalization. On hospitalization Day 7, blood tests showed no significant improvement, and the serum procalcitonin level was elevated at 0.057 ng/mL (normal range: 0–0.05 ng/mL). However, a follow-up chest CT revealed a slight reduction in the size of the round lesion in the right lower lobe (RLL) compared to the initial imaging (Figure 2B). The mNGS test identified a pulmonary infection caused by *N. aobensis.* Based on this diagnosis, the antibiotic regimen was adjusted to include TMP-SMZ in combination with imipenem on Day 10.

One week after the medication was adjusted, the patient reported a significant reduction in cough and sputum production, with occasional grayish sticky sputum and decreased intensity of chest pain. Repeat blood tests showed normalization of white blood cell count and CRP levels. On Day 18, chest CT showed further reduction in the size of the round lesion in the RLL, showing the formation of irregular cavities (Figure 2C). The patient’s clinical symptoms continued to improve, and she was discharged on hospitalization Day 24 with instructions to continue taking oral TMP-SMZ and linezolid for 6 months. The patient returned to the hospital for regular follow-up 1 month after discharge, reporting occasional chest tightness and dry cough, but no significant expectoration, fever, or wheezing. Chest CT showed a significant reduction in the size of the infectious lesion in the RLL, with no cavities observed (Figure 2D). The ESR was normal; however, blood tests showed a decreased white blood cell count (1.98 × 10^9^/L) and hemoglobin level (112 g/L), with a slightly elevated CRP level (6.190 mg/L). The patient also reported experiencing abdominal discomfort and nausea during the medication period after discharge. The decrease in white blood cells and hemoglobin was attributed to the side effects of the drugs. Therefore, the antibiotic regimen was changed to oral doxycycline for continued treatment. At a follow-up visit 4 months after discharge, the blood tests and CRP levels of the patient showed normal results. A chest CT showed further reduction in the lesion of the RLL compared to the findings at the first follow-up (Figure 2E). The most recent follow-up was conducted in April 2024 via telephone, and the patient’s condition remained favorable and exhibited a positive attitude toward the long-term medications.

## Discussion

*Nocardia* species are opportunistic pathogens that can infect the lungs, skin, central nervous system, and other organs, presenting as localized or disseminated infections. As inhalation is the primary route of bacterial exposure, pulmonary nocardiosis is the most common clinical presentation of nocardiosis. Because T-cell mediated immunity and lung macrophages play a key role in the local control of *Nocardia*, invasive nocardiosis mainly affects immunocompromised individuals (9). The most common predisposing factors for opportunistic nocardiosis are hematologic malignancy, human immunodeficiency virus infection, solid-organ or hematopoietic stem cell transplantation, and long-term immunosuppressive treatment with glucocorticoids (10, 11). Our patient had immune thrombocytopenia and had been on treatment with glucocorticoids for more than 2 years, which was a high-risk factor for *Nocardia* infection.

To the best of our knowledge, this is the first detailed case of pulmonary infection with *N. aobensis* detected by mNGS. The results of Sanger sequencing and conventional culture method also support the result of mNGS. *N. aobensis* was first identified from clinical isolates and characterized by Japanese scholar Kageyama et al. in 2004 (12). The infections caused by *N. aobensis* have rarely been reported in the literature. To date, only three cases of *N. aobensis* infection have been reported, of which two are detailed case reports involving skin and soft tissue infections, and the third one involves pulmonary infection in patients undergoing allogeneic hematopoietic stem cell transplantation, which was mentioned in a retrospective study but lacked a specific case description (13–15).

While *Nocardia* infections are not common in clinical practice, they carry a high mortality rate, reported to be between 18 and 30% for pulmonary nocardiosis. This rate is higher when *Nocardia* causes disseminated infections and brain abscesses (16). Currently, the clinical diagnosis of nocardiosis still depends on microbial culture. However, it usually takes 2–14 days for *Nocardia* to grow visible colonies, which can extend to 4–6 weeks for slow-growing species (10). The clinical symptoms of pulmonary nocardiosis are non-specific and can include fever, cough, expectoration, dyspnea, and chest pain. Chest radiographic findings are also variable, which may display focal or multifocal lesions with nodular, patchy, or consolidation shadows, and even cavities, masses, or pleural effusions (17). Thus, the time-consuming nature of *Nocardia* culture and the broad spectrum of clinical presentations pose significant diagnostic challenges for pulmonary nocardiosis. In addition, PCR and serology are culture-independent and convenient methods for identifying pathogens, but they are targeted detection methods that can only detect specific pathogens; therefore, the unexpected, rare or novel pathogens would be overlooked. *Nocardia* species are rare in clinical practice and are typically not the primary suspects in the identification of potential pathogens, which are frequently overlooked by clinicians. For the aforementioned reasons, pulmonary nocardiosis is often missed, diagnosed late, or misdiagnosed as other conditions such as bacterial pneumonia, tuberculosis, invasive fungal infection, or lung cancer, all of which significantly contribute to mortality. Thus, rapid and accurate identification of *Nocardia* in clinical samples is vital for early administration of appropriate antibiotic therapy, thereby reducing the mortality rate among patients with nocardiosis.

As an emerging molecular technology, mNGS is an unbiased, hypothesis-free, and culture-independent technology that can detect all microorganisms present in clinical samples in a single assay. It has considerable advantages, especially in the detection of rare, difficult-to-culture, or new pathogens (18). It can provide a faster detection turnaround time, higher accuracy, and better diagnostic performance compared to conventional methods. Its application in the early diagnosis of diseases has already been confirmed (19, 20). In 2020, an expert consensus on the clinical application of mNGS in the detection of infection pathogens was released in China. This consensus recommended that mNGS should be performed as a supplement to conventional etiological detection methods to identify rare pathogens or mixed infections as soon as possible for special patients such as immunosuppressive or immunodeficiency hosts, particularly in cases where conventional diagnostic assays are negative (21). In our case, the conventional methods, including the acid-fast staining of the sputum smear, bacterial and fungal cultures of the sputum, and serum aspergillosis galactomannan and (1,3)-*β*-d-glucan tests, were all negative. In addition, our patient did not respond to the initial empiric antimicrobial therapy for pneumonia well. Pulmonary infections are urgent and have a high mortality, with more complicated situations in old and immunocompromised patients (22, 23). In order to identify the pathogens as soon as possible, we applied mNGS to the BALF of the patient, then successfully detecting *N. aobensis.* The mNGS process, from submitting the sample to reporting the result, was completed in just 24 h. Meanwhile, traditional culture methods took 3 days longer than mNGS did and failed to specify the *Nocardia* species. In a recent retrospective study, the culture method had a positive rate of only 35.7% for *Nocardia*, an average turnaround time of 7.5 days, and it often failed to identify *Nocardia* at the species level. In contrast, mNGS achieved a positive rate of 100%, significantly reduced the turnaround time to 2 days, and accurately identified the *Nocardia* species involved (7). This showed the high sensitivity of mNGS in detecting *Nocardia*, its ability to differentiate between *Nocardia* species, and its potential to significantly reduce the turnaround time. A study had shown that immunocompromised patients, particularly those on long-term corticosteroid therapy, had a much higher mortality risk compared to both immunocompetent and immunocompromised patients not taking corticosteroids (24). Therefore, this advanced diagnostic approach enables prompt pathogen diagnosis and treatment, thereby improving patient outcomes. As antimicrobial sensitivity varies across *Nocardia* species, rapid and accurate identification of the specific pathogen at the species level is also crucial to guide clinicians in selecting the most effective initial antimicrobial treatment (25).

Trimethoprim-sulfamethoxazole has traditionally been the first choice for the empirical treatment of nocardiosis. Combination antibiotic therapy (generally the addition of amikacin and carbapenem) is preferred for immunosuppressed patients with localized or disseminated infections (26, 27). Studies have shown that *N. aobensis* is sensitive to TMP-SMZ, amikacin, imipenem, and linezolid (15). Therefore, we adjusted the antibiotic therapy of the patient to TMP-SMZ combined with imipenem, based on the results of mNGS. After 1 week of medication, her symptoms significantly improved, which indicated that the treatment was effective. It is recommended that the duration of treatment be 6 months for immunocompromised patients with localized infections to prevent the recurrence of nocardiosis (3). However, long-term treatment could be challenging due to the adverse events of antimicrobials. Although TMP-SMZ is a cornerstone of nocardiosis treatment, its long-term use is often limited by side effects such as nephrotoxicity, digestive disturbance, and bone marrow suppression (27). Similarly, linezolid, which is highly active against all known pathogenic *Nocardia* species, is expensive for prolonged use and can cause side effects including anemia, thrombocytopenia, and potential neurological adverse events and hematological toxicity (2, 28). The patient in this case was discharged with instructions to continue oral treatment with TMP-SMZ and linezolid for 6 months, and a follow-up CT scan 1 month later indicated that the treatment was effective. However, the patient developed adverse effects such as abdominal discomfort, nausea, vomiting, and decreased white blood cells and hemoglobin level. Therefore, the antibiotic regimen was switched to doxycycline, a better-tolerated tetracycline derivative. After 3 months of therapy adjustment, the patient returned to the hospital for another follow-up, without any adverse effects. Blood routine and CRP levels were normal, and chest CT showed that the lesion had further reduced, suggesting that doxycycline could be continued to complete the treatment course.

There are currently no standard recommendations for the treatment of nocardiosis. Treatment must be tailored based on the patient’s response to the initial treatment, clinical progression, and antimicrobial susceptibility testing. Although conventional culture and antimicrobial susceptibility testing are essential as the antimicrobial sensitivity varies by *Nocardia* species, we were unable to perform these tests due to the technical limitations of our clinical laboratory, which is the limitation of our case. *Nocardia* species were historically divided into groups based on their susceptibility patterns to various antimicrobial classes. If the results of susceptibility testing are not available, these group antimicrobial susceptibility profiles could be used as a rough guide to determine treatment regimens, although some of the groups show inconsistent patterns for some drug classes (1, 29). Because of the rarity of the case, there are no current guidelines on the treatment of *N. aobensis*, and therapy recommendations are based on expert opinions within our hospital. The characteristics of *N. aobensis* and effective treatment regimens require further investigation with the accumulation of more clinical cases. It is anticipated that mNGS will be widely used in the near future, leading to the detection of more cases of *N. aobensis* infection and the further accumulation of clinical experience.

## Conclusion

In conclusion, the clinical and radiological manifestations of nocardiosis are non-specific, making it challenging to diagnose through traditional culture methods which have low sensitivity and a high rate of false negatives. As a result, the diagnosis of nocardiosis is usually missed, delayed, or misdiagnosed. In this case, mNGS facilitated a rapid early-stage diagnosis of an infection with *N. aobensis* which is rarely reported in literature, allowing for timely adjustment of the antibiotic regimen. As a novel technology, mNGS offers better performance and efficiency compared to traditional methods in the diagnosis of infectious diseases, especially for rare and difficult cases. For immunocompromised patients presenting with non-specific symptoms and CT imaging results, the possibility of nocardiosis should be considered and mNGS should be performed as soon as possible to assist diagnosis and improve prognosis.

## Data Availability

The original contributions presented in the study are included in the article/supplementary material, further inquiries can be directed to the corresponding author.

## Footnotes

^1^ https://dataview.ncbi.nlm.nih.gov/object/PRJNA1154608
